# Stoma Detection in Soybean Leaves and Rust Resistance Analysis

**DOI:** 10.3390/plants14192994

**Published:** 2025-09-27

**Authors:** Jiarui Feng, Shichao Wu, Rong Mu, Huanliang Xu, Zhaoyu Zhai, Bin Hu

**Affiliations:** 1 College of Smart Agriculture (College of Artificial Intelligence), Nanjing Agricultural University, Nanjing 211800, China; 2 College of Engineering, Nanjing Agricultural University, Nanjing 211800, China; 3 Information Center of Qinghai Provincial Department of Agriculture and Rural Affairs, Xining 810008, China; 4 Agricultural Technology Extension Service Center, Xining 810008, China

**Keywords:** soybean rust, disease resistance, YOLOv8, attention mechanism, stomatal phenotypic parameters

## Abstract

Stomata play a crucial role in plant immune responses, with their morphological characteristics closely linked to disease resistance. Accurate detection and analysis of stomatal phenotypic parameters are essential for soybean disease resistance research and variety breeding. However, traditional stoma detection methods are challenged by complex backgrounds and leaf vein structures in soybean images. To address these issues, we proposed a Soybean Stoma-YOLO (You Only Look Once) model (SS-YOLO) by incorporating large separable kernel attention (LSKA) in the Spatial Pyramid Pooling-Fast (SPPF) module of YOLOv8 and Deformable Large Kernel Attention (DLKA) in the Neck part. These architectural modifications enhanced YOLOV8′s ability to extract multi-scale and irregular stomatal features, thus improving detection accuracy. Experimental results showed that SS-YOLO achieved a detection accuracy of 98.7%. SS-YOLO can effectively extract the stomatal features (e.g., length, width, area, and orientation) and calculate related indices (e.g., density, area ratio, variance, and distribution). Across different soybean rust disease stages, the variety Dandou21 (DD21) exhibited less variation in length, width, area, and orientation compared with Fudou9 (FD9) and Huaixian5 (HX5). Furthermore, DD21 demonstrated greater uniformity in stomatal distribution (SEve: 1.02–1.08) and a stable stomatal area ratio (0.06–0.09). The analysis results indicate that DD21 maintained stable stomatal morphology with rust disease resistance. This study demonstrates that SS-YOLO significantly improved stoma detection and provided valuable insights into the relationship between stomatal characteristics and soybean disease resistance, offering a novel approach for breeding and plant disease resistance research.

## 1. Introduction

Soybean [*Glycine max* (L.) *Merrell*] is one of the world’s most important food and oil crops. Its yield and quality are of great significance to global agricultural production and food supply. However, soybean cultivation is challenged by both biotic and abiotic stresses, with soybean rust being a primary concern. The global soybean planting area has reached 102.77 million hectares, with an annual production of 239.36 million tons [1]. Asian soybean rust (ASR) can infect more than 31 species across 17 genera of legumes, causing severe yield losses [2] and leading to annual global economic losses of 7-10 billion USD [3]. In China, ASR has been reported in 24 provinces. For breeders, identifying ASR-resistant soybean cultivars requires a clear understanding of trait associations, yet few studies have explored the relationship between biochemical and physiological variables, gas exchange components, and the occurrence of ASR [4]. Plant stomata serve as essential channels for plant–environment gas exchange, yet they also function as a principal pathway of pathogen penetration. Although stomata only comprise 0.3–5% of the leaf surface area, they account for 98% of gas exchange [5]. Plant stress triggers stomatal closure, resulting in vein embolism and disrupting water transport [6]. The morphology of guard cells and the density, size, and opening status of stomata are closely related to plant disease resistance [7,8]. Stomata regulate important physiological processes in plants, and researchers from different fields often conduct phenotypic analyses of stomata [9]. Accurate detection and analysis of stomatal characteristics can provide important biological evidence for revealing soybean rust resistance.

Traditional stoma detection methods rely on manual annotation and calculation by using ImageJ (version 1.54p), which is inefficient and labor-intensive [10,11]. The application of artificial intelligence to the analysis of stomatal characteristics enables an objective and accurate evaluation of physiological changes [12]. Therefore, the development of efficient and automated stoma detection technology is of great significance for revealing soybean disease resistance.

Artificial intelligence (AI) has been increasingly applied to predict growth rates and optimize irrigation, disease diagnosis, and pest detection [13]. In recent years, the rapid advancement of deep learning (DL) has brought new opportunities for plant image processing. For stoma detection, various DL models have been proposed [14,15,16,17]. Li [18] developed LeafNet, which employed a region-merging algorithm for segmenting Arabidopsis stomata and used a deep convolutional neural network to quantify 28 different morphological parameters, including stomatal number, size, and aspect ratio. StomataCounter [19] was proposed to detect stomata. However, it was observed that the image noise and edge issues may decrease the detection accuracy. Takagi [20] combined a YOLOX detection module and a U-Net stomata segmentation module to achieve quantification of the Arabidopsis stomatal diameter, with a stoma detection IoU of 0.875 and a stoma segmentation IoU of 0.745. Zhang [21] proposed DeepRSD for rotated maize leaf stoma detection by introducing a conductance loss function that can simultaneously detect rotated stomata and calculate basic stomatal traits. Casado-García [22] developed LabelStom, a stoma detection tool based on the YOLOv3 algorithm, for detecting stomata in common bean, barley, and soybean varieties. Yang [23] proposed a stoma detection framework combining feature-based transfer learning and YOLOv4, which achieved a detection accuracy of 0.994 for maize and wheat leaves while simultaneously calculating stomatal height and width. Pathoumthong [24] utilized the YOLOv5 model to detect stomata from non-destructively acquired images of wheat, rice, and tomato leaves and calculated parameters such as stomatal number and size. As deep learning models are highly dependent on training data, dataset quality directly affects model performance. Tomke [25] employed a U-Net segmentation model to analyze the stomata of wheat and faba bean, achieving high accuracy for stomatal density (R^2^ = 0.98) and size (R^2^ = 0.90). Yang [26] developed a novel lightweight detection model, StomaYOLO, tailored for small stoma targets and subtle features in microscopic images. The mean Average Precision (mAP) of StomaYOLO was improved by nearly 9% compared to YOLOv11. Shi [27] improved YOLOv8 by replacing the original Intersection over Union (IoU) calculation and incorporating Squeeze-and-Excitation (SE) and Self-Attention (SA) mechanisms. Furthermore, YOLOv8 achieved a detection accuracy exceeding 93% in studies of crops such as lettuce and wheat [28,29].

It is noted that the YOLO (You Only Look Once) family algorithms, due to their efficient, real-time, and high-precision characteristics, perform excellently in stoma detection and phenotypic calculation [30,31,32]. The YOLO series has undergone rapid evolution. YOLOv7 achieves strong performance on benchmarks, but its anchor-based design limits precision for small objects [33]. YOLOv9 improves accuracy with the GELAN backbone but introduces a higher computational cost and slower inference. YOLOv10 provides advantages in lightweight design and mobile deployment, yet its detection performance for small and densely packed objects in complex scenarios has not been systematically evaluated. In contrast, YOLOv8 employs an anchor-free structure with an optimized backbone, offering a balanced trade-off between accuracy and efficiency for small, densely distributed targets, such as soybean stomata. Therefore, YOLOv8 is selected as the baseline framework in this study. However, the detection of plant stomata still faces challenges such as low-clarity images, small object detection, and robustness in complex backgrounds. Especially when dealing with densely and irregularly distributed stomata in soybeans, YOLOv8 still requires further optimization to enhance its applicability in plant disease resistance analysis. Addressing this constraint requires further investigation into feature extraction, attention mechanisms, and multi-scale detection approaches.

**Figure 1:**
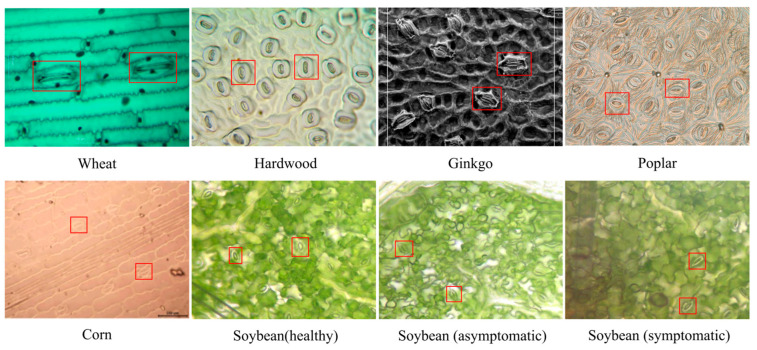
Stoma of different plant species. Note: The red boxes mark representative stomata, illustrating morphological differences across species.

In the stoma detection task, leaf characteristics vary widely across different plant species. For example, monocotyledonous plants like maize [34] and wheat typically have regular stomata with a relatively simple distribution pattern, making detection easier (Figure 1). However, research on leguminous crops, especially soybeans, remains limited [35]. Soybean leaves have villous surfaces, and their stomata are small and sparsely distributed [36,37]. It is worth noting that previous studies have employed a variety of microscopic imaging techniques, including nail polish imprinting, standard optical microscopy, camera-based imaging, and specialized microscopes. These techniques lead to varying stomatal visibility, contrast, and boundary clarity. In particular, soybean presents additional challenges due to its complex vascular structure, encompassing water and conductive tissues, which complicate accurate stomatal identification. Pathological changes in leaves during disease progression, such as chlorosis and lesions, make stoma identification and counting even more difficult. The complex background of soybean leaves, particularly the presence of texture interference and lesions, further reduces detection accuracy.

To address these challenges, this study proposed SS-YOLO, a deep learning model for soybean leaf stoma detection and stomata phenotypic parameter calculation. The goal was to improve the accuracy and efficiency of stoma detection and provide a new technical approach for soybean breeding and disease resistance research. The main contributions of this study are summarized as follows.

(1) A dataset of soybean leaf stomata was constructed, comprising healthy and rust-infected leaves from three varieties. The dataset contained 1800 RGB images with 25,396 labeled stomata instances.
(2) The SPPF module of the YOLOv8 backbone was enhanced via the Large and Separable Kernel Attention (LSKA) mechanism. LSKA weighted multi-scale feature maps, resulting in enhanced adaptation to variations in multi-scale feature maps.
(3) Deformable Large Kernel Attention (DLKA) was introduced into the C2f module of the Neck, adaptively capturing irregular shapes and sizes. This improved SS-YOLO’s robustness and detection performance in small object detection, complex backgrounds, and low-contrast images.
(4) Based on the detection results, phenotypic parameters like the stomatal length, width, number, density, area, and stomata index of soybean leaves were automatically extracted. The relationship between stomatal characteristics and disease resistance in different soybean varieties and at different disease stages was analyzed, demonstrating the potential impact of stomatal characteristics on disease resistance.

Through automated and accurate stoma detection, the efficiency of soybean disease resistance screening can be greatly improved, providing strong technical support for precision breeding. The rest of this paper is organized as follows. Section 2 introduces the data acquisition methods and dataset construction. Section 3 describes the establishment of the SS-YOLO model and the calculation of stomata phenotypic features. Section 4 analyzes the experimental results and the disease resistance of different soybean varieties. Section 5 discusses comparisons with other research work and the limitations of our work.

## 2. Materials and Methods

### 2.1. Plant Materials

- Plant Materials and Growth Conditions

Three soybean cultivars, Dandou 21 (DD21), Fudou 9 (FD9), and Huaixian 5 (HX5), were used in this study. Seeds were cultivated in a controlled greenhouse facility at Nanjing Agricultural University (Nanjing, China; 118°51′ E, 31°57′ N). Environmental conditions were maintained at 25–27 °C, relative humidity above 70%, and a daily photoperiod of 14 h light (05:00–19:00) and 10 h dark, with an average light intensity of 5000 Lux.

- Pathogen and Inoculum Preparation

The Asian soybean rust fungus (*Phakopsora pachyrhizi Syd.*) was provided by the GreenTechLab Co., Ltd. (Shanghai, China). Soybean leaves bearing visible uredinia were collected and placed in 2 L plastic containers. Spores were washed from leaf surfaces using 0.05% Tween-80 solution, shaken thoroughly, and filtered through sterile gauze. The resulting urediniospore suspension was adjusted to a concentration of 5 × 10^5^ spores/mL.

- Inoculation and Disease Management

Healthy soybean plants at the two-unifoliate leaf stage were uniformly inoculated with the spore suspension using a spray method. After inoculation, plants were transferred to a humidity chamber equipped with a centrifugal mist humidifier (Kangdi 3001, Condair Group, Pfäffikon, Switzerland) and maintained under dark conditions at 25–28 °C and 100% relative humidity for 24 h to promote spore germination and infection. To prevent cross-contamination through airborne dispersal, healthy and inoculated plants were cultivated in two separate greenhouses with identical environmental conditions. Each greenhouse was equipped with ceramic heating lamps, full-spectrum LED plant growth lamps, insect-trapping boards, timers, and temperature–humidity monitors [38].

- Preliminary Evaluation of Rust Resistance

**Figure 2:**
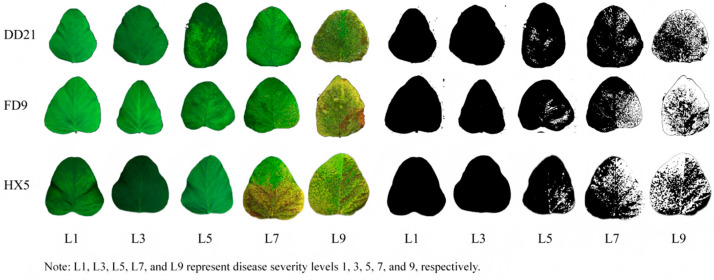
Time-series images of disease progression in various varieties.

To obtain a preliminary evaluation of the rust resistance of the examined cultivars, we recorded time-series images of disease progression in DD21, FD9, and HX5 (Figure 2). Visual inspection and quantification of lesion area indicated that DD21 developed fewer and smaller lesions across the entire infection period. In contrast, FD9 and HX5 showed faster lesion expansion and greater lesion coverage, reflecting weaker resistance. These observations provided a comparative basis for linking stomatal morphological traits with rust resistance performance in the subsequent analysis.

### 2.2. Collection Equipment and Methods

**Figure 3:**
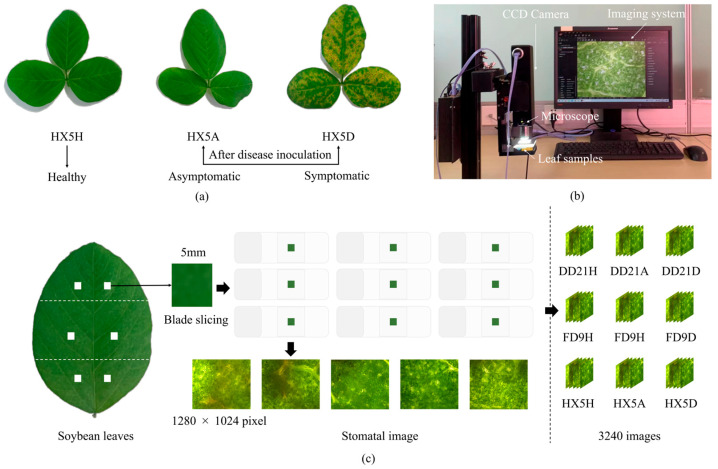
Data collection process. (**a**) Three varieties under healthy and infected status; (**b**) the Hikvision imaging system; (**c**) extraction of regions of interest. Note: DD21, FD9, and HX5 denote the soybean variety, while H, A, and D denote healthy, asymptomatic, and symptomatic, respectively. For instance, HX5H denotes the HX5 variety at the healthy stage.

To ensure consistency in data collection, stomata images were acquired daily at the same time (10:00–14:00), encompassing different leaf samples, namely healthy, asymptomatic (infected leaves without visible lesions), and symptomatic (infected leaves with visible lesions on the surface) (see Figure 3a). The stoma image acquisition equipment is shown in Figure 3b and mainly included a Hikvision Charge Coupled Device (CCD) camera, the Hikvision Machine Vision Software (MVS V4.5.1, Windows; Hangzhou Hikvision Digital Technology Co., Ltd., Hangzhou, China) industrial camera client software platform, an optical microscope (×20 magnification), a stand, a stage, an adjustable light source, and a computer. The trifoliate leaf structure of soybean was used, and each leaf was divided into three independent regions, including top, middle, and bottom, respectively. A 5 mm × 5 mm leaf sample was selected from each region, removed with tweezers, and placed on a glass slide. To ensure close contact between the leaf and the glass slide, sterile water was added, and a coverslip was placed over the sample. Subsequently, the glass slide was placed on the stage, the light source was adjusted to optimize image brightness, and the leaf was observed using an optical microscope at 20× magnification to ensure that the stoma images were clearly visible. Next, stoma images were captured from the clear field of view using the MVS. Stoma images were randomly captured from each region to ensure data randomness and discreteness. The images had a resolution of 1280 × 1024 pixels and were saved in BMP format, as shown in Figure 3c. Finally, a total of 3240 leaf stoma images were obtained from the three soybean varieties for subsequent experimental analysis.

### 2.3. Dataset Construction

Among the initial 3240 microscopic images, images with blur or poor focus that caused unclear stomatal boundaries, as well as images affected by water reflections, making stomatal features difficult to recognize, were excluded. In addition, images where leaf veins or vascular structures significantly obscured stomata were removed. Through this screening process, 1800 high-quality images were retained, which adequately represent the diversity of soybean leaf stomata under varying disease severity and ensured reliable and accurate model training and evaluation.

Each soybean variety had 600 images, covering three categories of samples: healthy, asymptomatic, and symptomatic. To ensure the accuracy of phenotypic parameter calculation, clear and complete regions of stomata were extracted from each image and cropped to a uniform size of 512 × 512 pixels. During the training process, the input images were adjusted to 640 × 640 pixels. Stomata were annotated by rectangular bounding boxes through the Roboflow platform https://app.roboflow.com/login (accessed on 1 September 2024). For incomplete stomata at the edges of the images, annotations were also marked if their area accounted for more than approximately two-thirds of the entire stomata. A total of 25,396 stomata were manually labeled. The dataset was randomly divided into a training set and a validation set by an 8:2 ratio. The details were listed in Table 1.

### 2.4. Stoma Detection Model SS-YOLO

**Figure 4:**
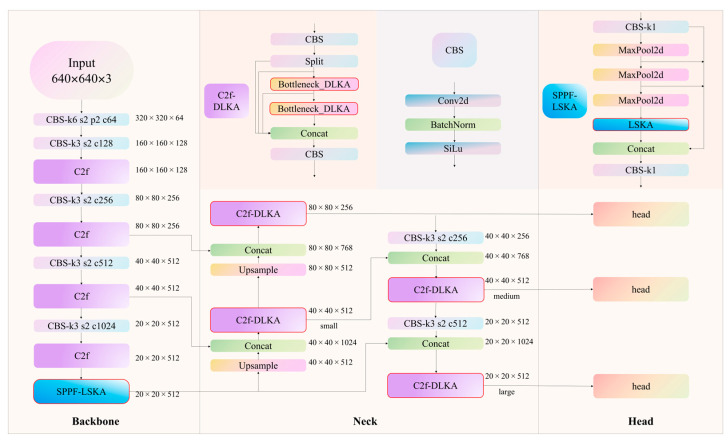
The architecture diagram of SS-YOLO. The red rectangular box marked the enhancements over the original YOLOv8 model.

To improve the accuracy and robustness of soybean stoma detection, this study proposed a deep learning model named SS-YOLO. The overall framework is shown in Figure 4. This study optimized the backbone and Neck parts of YOLOv8. In the backbone part, SS-YOLO introduced the SPPF-LSKA module. SPPF enhances robustness to small targets and low-contrast images through multi-scale pooling operations, while the LSKA module improves the attention to key areas through a large separable kernel attention mechanism. In the Neck part, the C2f-DLKA module further optimized feature fusion and refinement, enabling adaptive attention to stoma features. Through cross-layer fusion and up-sampling operations, the accuracy of stoma localization was further improved. Together, these architectural enhancements significantly improved the detection accuracy and reliability of SS-YOLO in the task of soybean stomatal identification.

### 2.5. SPPF-LSKA Module

**Figure 5:**
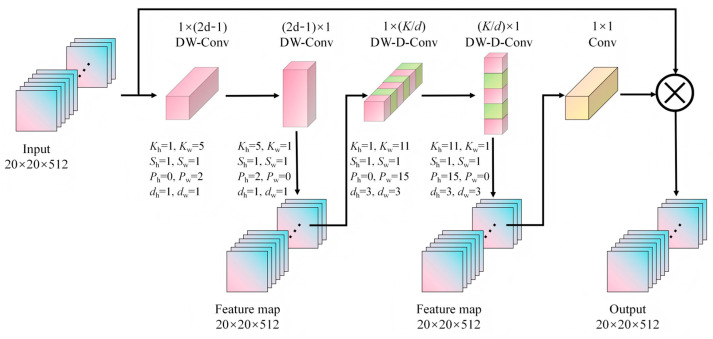
LSKA model architecture diagram. Note: Convolutional kernel, $K_{h}\times K_{w}$. Dilated convolution, $d_{h}\times d_{w}$. Stride, $S_{h}\times S_{w}$. Padding, $P_{h}\times P_{w}$.

The SPPF-LSKA module was designed to enhance multi-scale feature extraction and improve small object detection. The SPPF layer effectively aggregated features by performing max pooling operations at different scales. However, the original SPPF layer in YOLOv8 may have insufficient information extraction capabilities when dealing with complex scenes [39]. To address this issue, the LSKA mechanism was introduced to further improve the efficiency and accuracy of feature extraction. The LSKA structure (see Figure 5) was constructed by standard depthwise convolution (DW-Conv), dilated depthwise convolution (DW-D-Conv), and 1 × 1 convolution layers [40]. In LSKA, the first two layers of LKA were decomposed into four layers, each consisting of two 1D convolutional layers, where K represents the maximum receptive field, and d represents the dilation rate.

The LSKA mechanism captured the contextual information from images by using large and separable convolution kernels. Firstly, a *K × K* convolution was decomposed into (2d − 1) × (2d − 1) depthwise convolution, K/d × K/d depthwise dilated convolution, and 1 × 1 convolution. Secondly, the 2D depthwise convolution kernel and the depthwise dilated convolution kernel were further decomposed into 1D horizontal and vertical convolution kernels, and finally, the decomposed convolution kernels were concatenated sequentially [41]. Specifically, we first adopted DW-Conv (i.e., 1 × (2*d* − 1) and (2*d* − 1) × 1) operators, where *d* = 3, resulting in sizes of 1 × 5 and 5 × 1. The stride and padding were set to 1 and 2, respectively. Then, DW-D-Conv operators were employed with a kernel size of 11. At this time, the stride and padding were set to 1 and 3, respectively. We also used a 1 × 1 convolution operator before merging the feature maps. The detailed parameter settings are presented in Figure 4.

In the soybean stoma detection task, the SPPF module enhanced SS-YOLO’s perception of feature maps of different sizes, while the LSKA module improved the expressive power of stomatal features through the attention mechanism. This allowed the network to focus more on the key information, such as stomatal edges, thereby improving the detection accuracy.

### 2.6. C2f-DLKA Module

The Neck part of YOLOv8 used the C2f (Cross-Stage Partial Fusion) module multiple times, which was a crucial feature fusion module. Its main purpose was to improve feature extraction and the receptive field while obtaining a rich gradient information flow [42]. The C2f-DLKA module optimized the C2f structure by replacing the standard convolution operator with DLKA (Deformable Large Kernel Attention). Especially when dealing with small soybean stomata, DLKA enhanced the model’s adaptability to feature maps of different scales.

The C2f module initiated with a convolution layer. The resulting feature maps subsequently traversed through n Bottleneck modules. These features were concatenated and processed by a convolution layer to yield the final output. The DLKA module employed the deformable convolution operator and a large convolution kernel design, enhancing the adaptive processing capability for stoma features of varying sizes. Traditional convolution operations use fixed-size convolution kernels [43]. In contrast, the DLKA module adopted deformable convolution kernels, which dynamically adjust their sampling points.

The standard convolution kernel has a fixed size and shape. Therefore, for any point $P_{0}$ on the input feature map, the convolution operation can be represented by Equation (1):

(1) $$y\left(P_{0}\right)=\sum_{P_{N}\in R}w\left(P_{n}\right)\;\times \;x(P_{0}+P_{n})$$

where $P_{0}$ represented the offset of each point in the convolution kernel relative to the center point, $w(P_{n})$ represents the weight of the convolution kernel at the corresponding position, $x(P_{0}+P_{n})$ represents the element value at position $P_{0}+P_{n}$ on the input feature map, and $y(P_{0})$ represents the element value at position $P_{0}$ on the output feature map, which was obtained by the convolution operator.

An additional convolution operation was employed to learn offsets from the feature maps. These learned offsets were used to shift the sampling locations of the convolution kernel on the feature maps. The deformable convolution incorporated these offsets into the aforementioned formula, as shown below:

(2) $$y(P_{0})=\sum_{P_{N}\in R}w(P_{n})\times x(P_{0}+P_{n}+\Delta P_{n})$$

where $\Delta P_{n}$ represents the offset.

**Figure 6:**
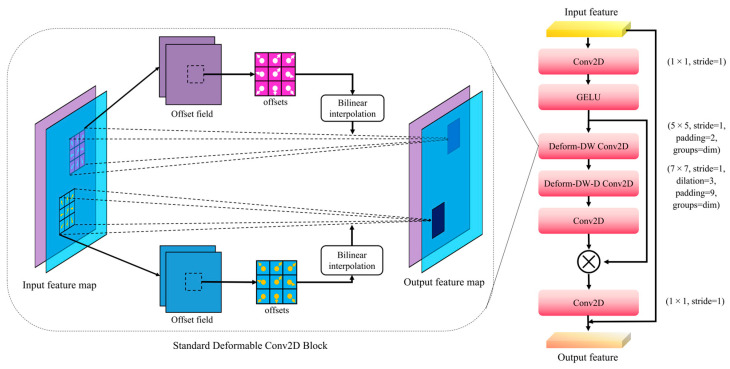
The architecture diagram of the DLKA module.

The structural implementation of DLKA is illustrated in Figure 6. First, the input feature map was processed by a standard convolution operation (Conv2D), followed by dynamic adjustment of features through deformable convolution. SS-YOLO further employed the depthwise separable convolution (depthwise convolution) and deformable depthwise convolution (Deform-DW2D Convolution) to enhance the non-linear expression ability. Subsequently, the convolutional output was normalized and transferred to the activation function. By employing depthwise separable convolution (DW) and depthwise dilated convolution (DW-D), SS-YOLO can learn features within a larger receptive field while simultaneously reducing the computational load by decreasing the number of parameters.

Specifically, we first employed a standard 1 × 1 convolution operator with a stride of 1 to compress the input feature channels, followed by a GELU activation function. Then, a deformable depthwise convolution with a 5 × 5 kernel, stride of 1, and padding of 2 was applied to extract local spatial features while enabling adaptive receptive fields. After that, a dilated deformable depthwise convolution was employed with a kernel size of 7 × 7, dilation = 3, stride of 1, and padding of 9, which enabled the module to capture long-range spatial dependencies. Subsequently, another 1 × 1 convolution was used to fuse the feature maps obtained from the deformable branches. Finally, the resulting attention map was multiplied by the shortcut input and passed through a 1 × 1 convolution to generate the output features. The detailed parameter settings are illustrated in Figure 6.

### 2.7. Calculation of Stomatal Characteristics

The leaf surface of plants serves as the primary line of defense against external pathogens, with the structure and function of stomata directly influencing the invasion pathways of these pathogens [44]. Consequently, the phenotypic characteristics of stomata, to some extent, determine the disease resistance of plants. Studies have shown that stomatal density, distribution, and behavior are all significant factors in the plant’s defense mechanisms against diseases [20,45]. Many pathogenic fungi exploit stomata and affect their movement, with rust fungi, in particular, evolving specific mechanisms targeting leaf epidermal structures such as guard cells [46].

**Figure 7:**
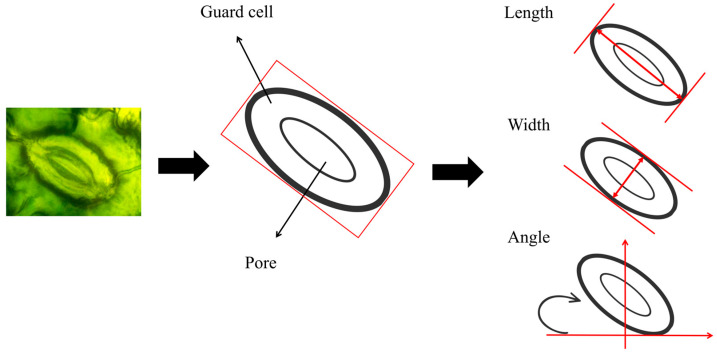
Calculation of stoma characteristics.

To further investigate the relationship between stomatal characteristics and plant disease resistance, Table 2 quantifies multiple physiological and morphological features of stomata, including basic parameters, morphological parameters (Figure 7), and the stomatal index [47]. This quantitative information provides an in-depth analysis of stoma function in plants under different disease states and reveals their performance in response to external environmental changes. Through comprehensive analysis of these parameters, we aimed to evaluate the role of stomata in health states and disease resistance.

### 2.8. Model Training and Evaluation Indicators

During training, the batch size was set to 4. The model utilized the Stochastic Gradient Descent (SGD) optimizer with an initial learning rate of 0.01 and a momentum parameter of 0.937 to accelerate gradient updates, thereby improving the training speed and convergence stability. Furthermore, a weight decay coefficient of 0.0005 was set to prevent overfitting. All input images were first resized to a uniform resolution of 640 × 640 pixels and normalized to the [0, 1] range to ensure training stability and facilitate convergence. To enhance SS-YOLO’s generalization ability, various data augmentation techniques were employed during training, including mosaic augmentation (with an intensity of 1.0), which increased data diversity by concatenating four images into a new composite image. Image translation (with a range of 0.1) and scaling (with a range of 0.5) operations further simulated variations in image scale and position. Image translation changes the stoma’s position in a given image. A range value of 0.1 indicates that the image was randomly translated by a value chosen uniformly from −0.1 to 0.1 on the x-axis and another independent random value from the same range on the y-axis. The scaling operation augmented the image by adjusting its scales, and the scaling factor indicated the magnification levels.

The training task was executed on an NVIDIA GeForce RTX 3090 GPU, utilizing CUDA acceleration for computation to enhance training speed and memory efficiency. To better manage computational precision and resource consumption during training, automatic mixed precision (AMP) was enabled. The entire training process was completed within 150 epochs. After each epoch, the detection performance was evaluated on the validation set.

We employed *Precision*, *Recall*, and mean Average Precision (*mAP*) as evaluation metrics for assessing the model’s detection performance. The calculation methods for Precision, Recall, and mAP are shown in Equations (3)–(5):

(3) $$Precision=\frac{TP}{FP+TP}$$

(4) $$Recall=\frac{TP}{TP+FN}$$

(5) $$mAP=\frac{1}{N}\sum_{i=1}^{N}{AP}_{i}$$

To comprehensively evaluate the performance of the proposed models, three widely used metrics in computer vision are adopted, namely, Frames Per Second (*FPS*), Parameters (*Params*), and Giga Floating-Point Operations (*GFLOPs*). The equation for calculating FPS is given as follows.

(6) $$FPS=\frac{N}{T}$$

where N denotes the number of processed images, and T represents the elapsed inference time in seconds. A higher FPS indicates faster inference speed and better real-time capability on a given hardware platform.

The number of parameters quantifies the storage size and structural complexity of a model, usually expressed in millions (M). Evaluating the number of parameters is a common method for measuring the strength of a model, and by fine-tuning the number of parameters, a balance between model efficacy and computational efficiency can be achieved. GFLOPs indicate the number of floating-point operations required to process a single input image, measured in 10^9^. A larger GFLOPs value reflects higher computational complexity, leading to increased inference latency and energy consumption.

In summary, FPS reflects real-time inference efficiency, Params measure model size and storage requirements, and GFLOPs characterize computational complexity. Together, these three indicators provide a comprehensive evaluation of model performance across different application scenarios.

## 3. Results

### 3.1. Ablation Experiment

The ablation experiment aimed to evaluate the impact of different modules on detection performance, and the results are presented in Table 3. By progressively modifying the key components of the models, the contribution of each module to the final detection performance was analyzed.

Table 3 shows that LSKA improved Recall and mAP_50_, reflecting enhanced multi-scale feature adaptation. DLKA further improved Precision and Recall, indicating stronger capability in detecting irregular and low-contrast stomata. When both modules were applied, the model achieved the highest overall performance while maintaining a reasonable inference speed and computational efficiency, demonstrating the joint effect of the two modules. SS-YOLO achieved superior performance across all metrics. Notably, it achieved 0.987 and 0.854 in mAP_50_ and mAP_50:95_, respectively, surpassing other models. SS-YOLO achieved 0.959 in Precision and 0.950 in Recall, indicating its strong capability to accurately detect stomata while effectively minimizing both false positives and false negatives.

**Figure 8:**
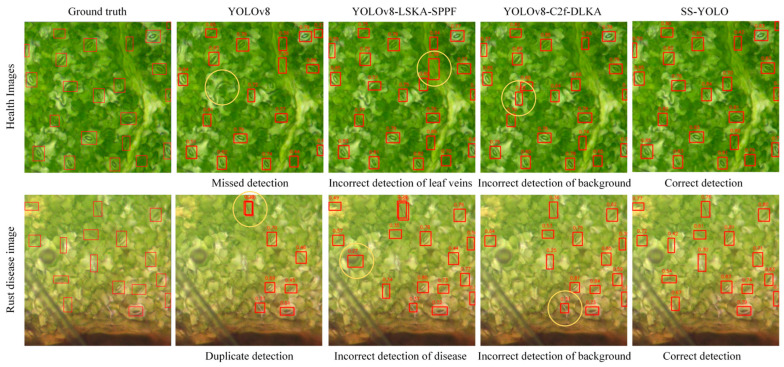
Stoma detection results of different models. Red boxes indicate detected stomata, while yellow circles, shown as examples, denote false positives, allowing an intuitive comparison of detection performance across models.

To provide a more intuitive comparison of the detection performance, Figure 8 illustrates the detection results of different models. The detection results of the original YOLOv8 model showed missed detection, with some stomata being unidentified and incorrectly identified as background. The YOLOv8-LSKA-SPPF model showed enhanced detection accuracy, but there were still misidentified instances. The YOLOv8-C2f-DLKA model showed slight improvement compared to the previous two, with some alleviation of missed detection and incorrect identifications. SS-YOLO exhibited the best detection results. It not only accurately identified all stomata but also had generally high confidence scores for most detection results. This indicates that SS-YOLO can provide more accurate detection results when dealing with the complex backgrounds of soybean leaves.

### 3.2. Comparative Experiments with Mainstream Models

To comprehensively demonstrate the advantages of SS-YOLO, we compared it with the YOLO family models and the state-of-the-art (SOTA) models (Table 4). First, YOLOv7, YOLOv9, and YOLOv10, as popular object detection models, were widely deployed in agricultural practice. The comparison results between the SS-YOLO model and SOTA models, ASF-YOLOv8 [48], DynamicCov-YOLOv8 [49], and StomaYOLO [26].

As shown in Table 4, SS-YOLO outperformed all compared SOTA models across key evaluation metrics, achieving a Precision of 0.959 and an mAP_50:95_ of 0.854, representing improvements of 18.3% and 5.3% over YOLOv7 and YOLOv10, respectively. Compared with other YOLOv8 variants such as ASF-YOLOv8, DynamicCov-YOLOv8, and StomaYOLO, SS-YOLO simultaneously increased Precision by 1.8–3.4% and Recall by 2.4–10.0%, while maintaining an inference speed of 34.03 FPS. These results indicate that SS-YOLO not only preserves the structural advantages of YOLOv8 but also achieves superior detection accuracy, robustness, and practical applicability through architectural optimizations.

**Figure 9:**
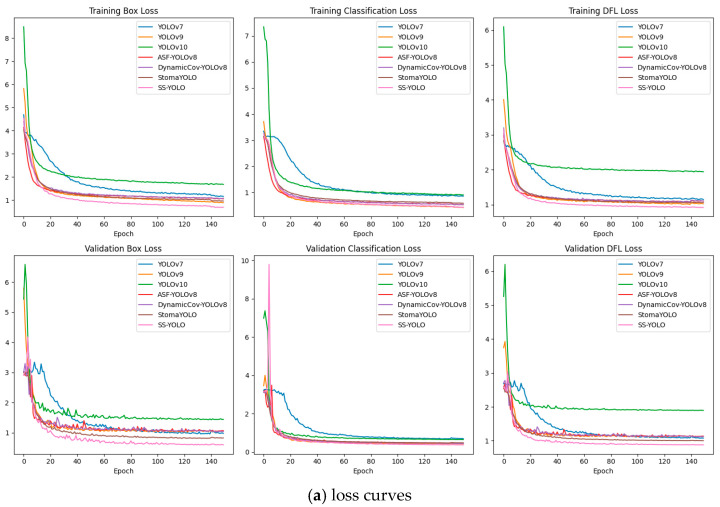

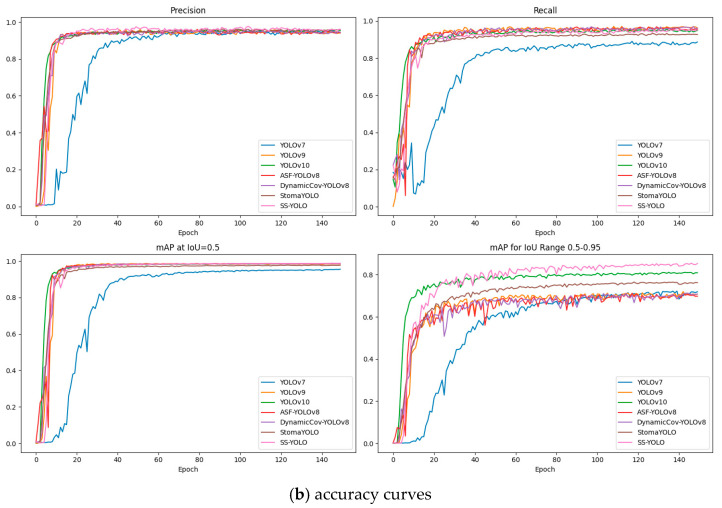
Loss and accuracy curves of all models.

To intuitively demonstrate the training process of each model, the loss function curves in Figure 9 evaluate the convergence of each model. During the training process, the training loss of SS-YOLO (including Box Loss, Classification Loss, and DFL Loss) converged significantly faster than others. In addition, the validation loss curve of SS-YOLO exhibited smaller fluctuations compared to the YOLO family and SOTA models, indicating that SS-YOLO had better stability on the validation set. Simultaneously, from the accuracy curve, it can be seen that SS-YOLO maintained high accuracy and Recall throughout the training process.

### 3.3. Model Generalization Experiment

**Figure 10:**
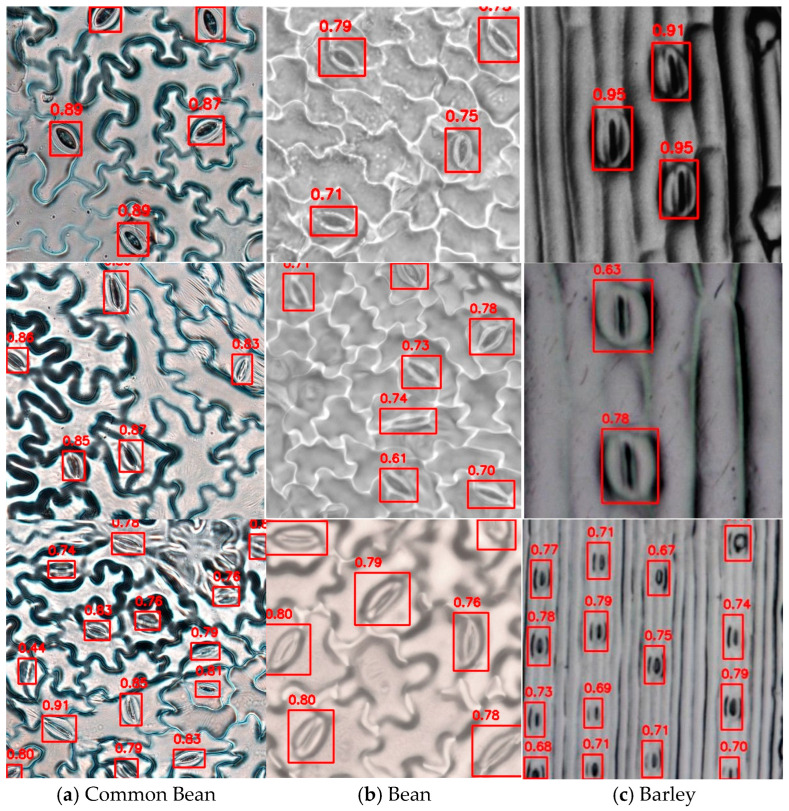
Stoma detection results on the public dataset. Note: the red boxes indicate detected stomata, and the numbers represent the confidence scores.

To evaluate the generalization performance of SS-YOLO, experiments were also conducted on a public dataset [22]. The public dataset covered stoma images of dicotyledonous plants (common bean, *Phaseolus vulgaris*; soybean, *Glycine max*) and monocotyledonous plants (barley, *Hordeum vulgare*). The common bean, soybean, and barley datasets contain 5400, 3990, and 8460 images, respectively. All images had a resolution of 400 × 400 pixels. This provided diverse test scenarios for evaluating the detection performance of SS-YOLO under different species. Figure 10 illustrates the detection results of SS-YOLO on the publicly available datasets of common bean, soybean, and barley.

The experimental results in Table 5 show that SS-YOLO exhibited strong detection capabilities over different plant species. In the public dataset, the common bean dataset performed better than the bean and barley datasets, with mAP_50_ and mAP_50:95_ reaching 0.988 and 0.751, respectively. The barley dataset exhibited comparatively lower Recall (0.898) and mAP_50:95_ (0.610), potentially attributable to the visual similarity between barley stomata and leaf vein patterns.

Overall, the experimental results validated the generalization ability of SS-YOLO on diverse datasets. This demonstrates that SS-YOLO can not only efficiently adapt to stoma detection tasks in different plant species and complex backgrounds but also provides research directions for further optimizing the model to improve its performance in scenarios with sparse targets and monocotyledonous plants.

### 3.4. Stomatal Characteristics Calculation Results and Disease Resistance Analysis

#### 3.4.1. Stomatal Characteristics Calculation Results

**Figure 11:**
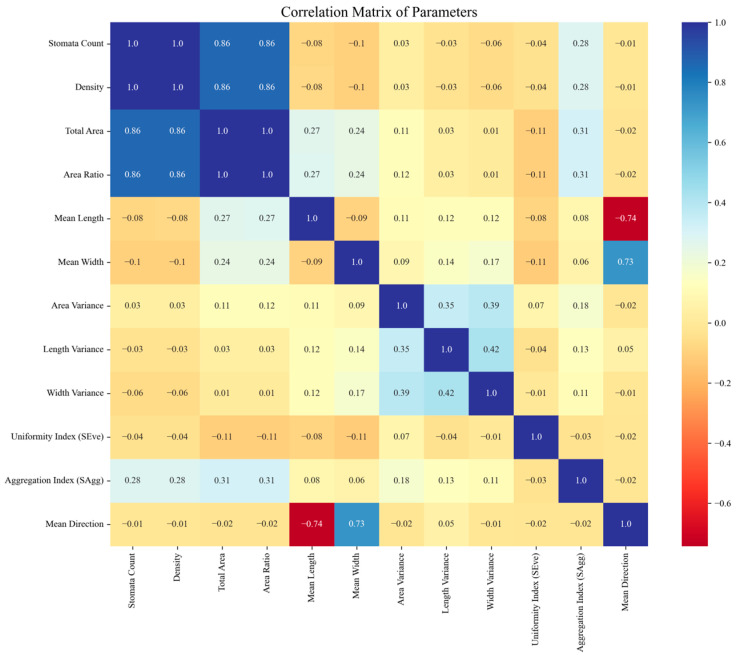
Correlation matrix between all stomatal characteristics.

The correlation matrix of all stoma features is shown in Figure 11. As can be seen, stomata count had strong positive correlations with density, total area, and area ratio, with correlation coefficients of 1.00, 0.86, and 0.86, respectively. This indicates that the increase in stomata count was usually accompanied by an increase in stoma density and total area, which was a natural phenomenon in the stoma distribution pattern. The aggregation index (SAgg) had strong correlations with total area and density, with correlation coefficients of 0.31 and 0.28, respectively. The aggregation index was related to the density of stoma distribution. The denser the stoma distribution, the higher the aggregation. The uniformity had a low correlation with other parameters, indicating that the stoma distribution in this dataset was relatively uniform.

We performed a comparative analysis of the stomatal parameters, including density, total area, length, width, and orientation, across three soybean varieties (DD21, FD9, and HX5) under varying health conditions (healthy, asymptomatic, and symptomatic). The statistics summary of the stomatal parameters is documented in Table 6. By comparing the stomatal characteristics of different varieties and health states, it can be found that the DD21 variety is superior to the FD9 and HX5 varieties in terms of stomatal density, total area, and stomatal length and width. Especially in the healthy and asymptomatic stages, the stomatal density and total area of DD21 were significantly higher than those of the other two varieties, which may have stronger stomatal function, consistent with its stronger disease resistance. For example, the stomatal density of DD21 in the healthy stage was 70, much higher than 55 for FD9 and 67 for HX5. This showed that DD21 has a denser distribution of stomata, and this feature may make it more advantageous in coping with diseases. Data analysis revealed a trend of decreasing stomatal density and total area with disease progression within each soybean variety. Conversely, stomatal width exhibited an increase as the disease advanced.

Through a comparative analysis of the stoma feature calculation results, we can not only identify the differences in stoma characteristics among varieties but also gain a deeper understanding of the potential relationship between stoma structure characteristics and soybean disease resistance. These results provide an important reference for future disease resistance evaluation and variety improvement.

#### 3.4.2. Analysis of Stomatal Parameters Among Different Soybean Varieties

**Figure 12:**
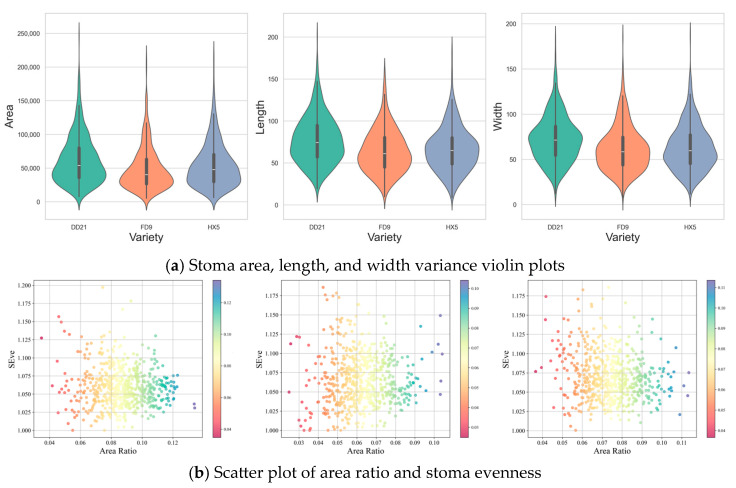
Stomatal parameter analysis of different soybean varieties.

The variations in stomatal parameters and disease resistance among different soybean varieties (DD21, FD9, and HX5) are illustrated in Figure 12. Specifically, violin plots (see Figure 12a) depict the variance in stoma area, length, and width across the three varieties. The horizontal axis represents varieties, and the vertical axis denoted scores (percentage). The shape of the violin plots reflects data density, with wider sections indicating higher data concentration and narrower sections indicating lower data concentration. Across the three variance plots, the score distribution of DD21 is consistently concentrated around 50%. For FD9, scores are distributed from 20% to 80% in the area and width variance plots, while the score distribution in the length variance plot centers around 50%, slightly lower than DD21. HX5 exhibits a score distribution primarily between 40% and 80% in the area and width variance plots, whereas the length score distribution is dispersed, ranging from 20% to 80%. These results suggest that DD21 demonstrates relatively consistent and stable score performance across all parameters.

The relationship between the area ratio and the stomatal evenness index (SEve) is shown in Figure 12b. The color of each point represents the size of the area ratio, reflecting the change in the area ratio, and the distribution of the points shows the correlation between the two. Data points with larger area ratios are usually associated with points with higher evenness indices, indicating that the increase in the area ratio may be related to the uniformity of the stomata. The DD21 variety had a more uniform stomatal distribution (SEve concentrated between 1.02 and 1.08) and a stable area ratio (0.06–0.09), which may help improve the gas exchange efficiency of stomata and reduce the chance of pathogen invasion, thereby exhibiting strong disease resistance. The FD9 variety had a more dispersed stomatal distribution (SEve mostly below 1.02) and a wider range of area ratio distribution (0.03–0.10). The HX5 variety had large fluctuations in stomatal distribution (SEve from 1.00 to 1.15), and the distribution of the area ratio was also more dispersed (0.03–0.11), which may lead to instability in the stomatal regulation function.

A statistical analysis of the stomatal parameters indicates that the DD21 variety exhibits higher uniformity in stomatal distribution, a stable area ratio, and smaller variances (area, length, and width) with concentrated distribution. These characteristics suggest that its stomatal morphology is stable and has strong disease resistance.

#### 3.4.3. Analysis of Stomatal Parameters at Different Disease Stages

**Figure 13:**
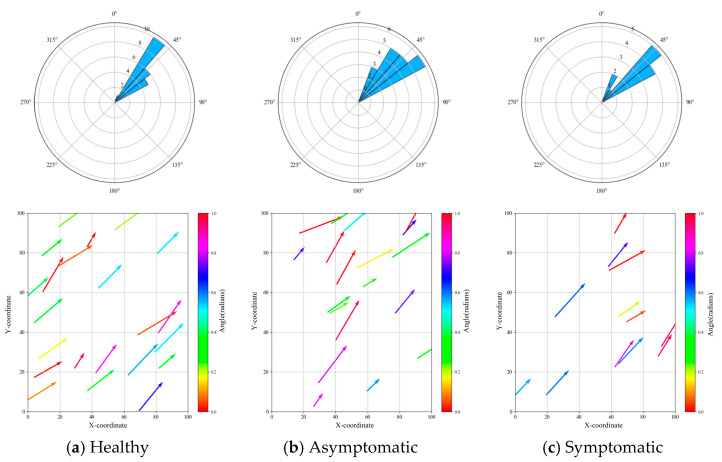
Analysis of soybean stomatal parameters under healthy and infected conditions.

A comparative analysis of the directional distribution and length variation in stomata during healthy, asymptomatic, and symptomatic states is presented in Figure 13. The rose diagrams show the distribution of stoma orientation in each stage. The arrow diagrams show the relationship between the orientation and length of the stomata. It can be found that the stomatal orientation distribution of healthy leaves is more diverse, concentrated in the range of 45–90°. The diverse stomatal distribution may help plants maintain normal gas exchange and transpiration. The distribution range of stomatal orientation narrowed in the asymptomatic period, mainly concentrating in specific directions (e.g., around 45°). It showed that although the plant had no visible symptoms, it may have reduced the invasion of pathogens by regulating the opening and closing of stomata. This was possibly an early defense response. The stoma orientation in the symptomatic period was more singular. It showed that the stoma function was impaired, which may be the result of the plant reducing diffusion by closing the stomata.

These changes reflect the impact of soybean disease development on stoma distribution and morphology. Healthy plants exhibited strong stoma diversity, while infected plants showed a significant decline in stoma function [50]. This suggests that with the development of soybean disease, the distribution characteristics and morphology of stomata undergo significant changes, which may affect their adaptability to environmental changes and disease resistance.

#### 3.4.4. Analysis of Stomatal Parameters and Disease Resistance

**Figure 14:**
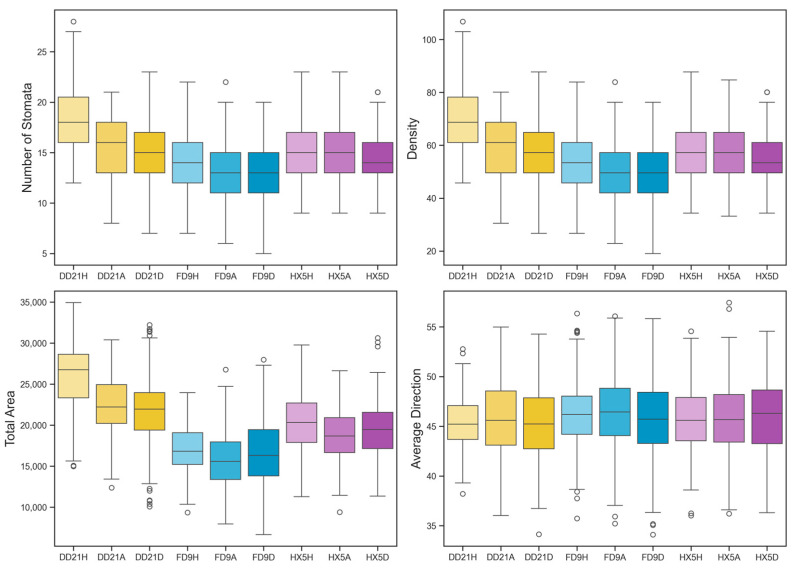
Analysis of stomatal parameters of soybean in different varieties at different stages of dyeing disease.

Stoma number, density, area, and orientation of soybean varieties under different disease states are significantly different, as depicted in Figure 14. First, in the healthy state, the DD21 variety exhibited high stability in stomatal number, density, area, and orientation. The median stomatal number and density of DD21H were the highest among the varieties, suggesting robust disease resistance, while FD9H and HX5H had lower stomatal numbers and densities, a wider distribution range, and greater volatility. After entering the asymptomatic state, the stomatal number, density, and area of DD21 slightly decreased, but the distribution remained concentrated, indicating its strong adaptability to diseases. The stoma number and density of FD9 and HX5 did not change much, but the degree of dispersion increased, indicating that they may have been under certain disease stress at this stage, but did not significantly affect their stoma function. In the diseased state, the DD21 variety showed the smallest variation in stoma number, density, area, and orientation, while FD9 and HX5 showed a significant decrease in stoma number, density, and area in the diseased state, with increased volatility, indicating that they had weak resistance to diseases, and their stoma function was greatly affected.

Specifically, in terms of stoma number, the DD21 variety maintained a relatively small variation between 10 and 22 in all states, showing its good stability. Its ability to maintain a relatively stable number of stomata under disease stress means that this variety can maintain good physiological functions and defense capabilities under disease infection. In contrast, the FD9 variety had a stoma number variation range between 6 and 18 in the healthy (FD9H) and asymptomatic (FD9A) states, and the stoma number varied from 5 to 20 in the symptomatic (FD9D) state. The HX5 variety showed a similar trend. The fluctuation in the number of stomata was large, ranging from 5 to 18, and the stability was poor.

In terms of stomatal density, DD21 maintained the highest density across all states, and HX5 showed the lowest, with stomatal density gradually decreasing as plant health declined. Specifically, the stomatal density of DD21 in the healthy state (DD21H) was about 70 stomata/μm^2^, slightly decreasing to about 59 stomata/μm^2^ in the asymptomatic state (DD21A), and further declining to approximately 57 stomata/μm^2^ in the diseased state (DD21D), representing a decrease of 18.6%. In contrast, the density of FD9 decreased from 55 stomata/μm^2^ (FD9H) to 48 stomata/μm^2^ (FD9D), a reduction of 12.7%, while HX5 decreased from 58 stomata/μm^2^ (HX5H) to 54 stomata/μm^2^ (HX5D), a decrease of 6.9%. The relatively rapid decrease in stomatal density observed in DD21 during disease progression may reflect an active defense response that helped limit pathogen invasion, with its dynamic structural regulation playing a key role in disease resistance mechanisms. Thus, stomatal density and its dynamic changes reflected the plant’s defense strategies by modulating the stomatal structure to restrict pathogen entry.

In terms of stoma area, the FD9 variety had the largest stoma area, with the stoma area in the healthy (FD9H) state being approximately between 12,000 and 35,000 μm^2^, with large variations, showing its instability in the stomatal area. In the asymptomatic (FD9A) and symptomatic (FD9D) states, the stomatal area varied in a wide range, indicating that the stomatal area of this variety was not stable enough, which may affect its overall physiological function and disease resistance. In contrast, the DD21 variety had a relatively small stomatal area and small fluctuations, maintaining between 15,000 and 25,000 μm^2^, showing high stability, which further supported its strong disease resistance. The stomatal area of the HX5 variety was similar to that of FD9, also showing large fluctuations, especially in the symptomatic (HX5D) state, where the change range of the stomatal area was large, showing its weak disease resistance.

Regarding stomatal orientation, the DD21 variety maintained a relatively consistent stomatal orientation in all states. Even in the symptomatic (DD21D) state, the change in stomatal orientation was still small, indicating that this variety can maintain its stomatal orientation and function well under disease stress, which was conducive to its disease resistance. In contrast, the stomatal orientation of the FD9 and HX5 varieties fluctuated greatly, especially in the symptomatic (FD9D and HX5D) states, showing low stability. This may indicate that the physiological functions of stomata in these varieties were greatly affected when dealing with diseases, and their disease resistance was weak.

Although the stomatal density of DD21 decreased considerably during disease progression, its total stomatal area remained higher than that of other cultivars at all disease stages, indicating that stomata can rapidly adjust their morphology and function under pathogen stress [7,51]. Despite the decline in density, the maintenance of total area ensured adequate gas exchange in leaves, helping to limit pathogen invasion while sustaining photosynthesis and metabolic activity. Additionally, this stomatal structure reduced local humidity accumulation, thereby decreasing spore germination and infection success. Overall, through dynamic regulation of stomatal traits, DD21 exhibited significant physiological resistance during early disease stages. The differences in stomatal parameters further demonstrated that DD21 maintained advantages in stomatal number, density, total area, and orientation, reflecting its strong disease resistance and physiological adaptability. FD9 exhibited intermediate performance, while HX5 showed inferior values in multiple parameters. Stomatal characteristics can serve as key physiological markers of rust resistance potential, providing an important basis for disease resistance evaluation and screening of different soybean varieties.

## 4. Discussion

### 4.1. Strengths of SS-YOLO

SS-YOLO enabled automated soybean stoma detection and the precise calculation of phenotypic parameters, significantly improving efficiency and saving labor costs compared to manual counting [30]. Through multi-dimensional technological innovations, SS-YOLO demonstrated outstanding performance in soybean stoma detection and cross-species adaptability. Firstly, the optimization of the model architecture significantly enhanced feature learning capabilities: in the backbone network, the SPPF-LSKA module addressed the feature response deviation caused by scale differences in stoma images (e.g., leaf folds or uneven illumination) through dynamically weighted multi-scale feature maps. Secondly, the C2f-DLKA module enhanced the parsing ability of low-contrast stomata edges and irregular shapes through adaptive convolution kernel deformation and collaborative optimization of local and global features. Experimental results showed that SS-YOLO achieved a detection accuracy of 98.7% on the self-built soybean stoma dataset, which was 3.4%, 10%, and 5.1% higher than the baseline model YOLOv8 in terms of Precision, Recall, and mAP, respectively.

Furthermore, SS-YOLO exhibited remarkable robustness in cross-species generalization validation. Testing results on the public datasets of common bean, bean, and barley demonstrated that the SS-YOLO model achieved an average detection accuracy of 96.2% on these datasets. Despite significant differences in guard cell arrangement and epidermal structure between monocotyledons (e.g., barley) and dicotyledons (e.g., soybean and common bean), SS-YOLO effectively maintained high-precision detection through a multi-scale feature decoupling strategy. This result verified that SS-YOLO can maintain excellent detection performance when facing stoma morphology and diverse characteristics of different species. The strong generalization ability provided a reliable tool for cross-species research on plant physiological phenotypes. This model can be extended to monitor leaf diseases and analyze disease resistance phenotypes in other crops.

Finally, SS-YOLO provided a new development tool for precision agriculture research. Quantitative analysis of stomatal parameters (e.g., density, size, area, and orientation) can provide important evidence for crop disease resistance evaluation. For example, high-throughput screening of the negative correlation between stoma density and pathogen infection resistance can accelerate the process of disease-resistant breeding. However, infection by different pathogens may lead to variations in stomatal morphology, which could affect detection accuracy and reliability. Moreover, achieving real-time monitoring may still rely on high-throughput imaging platforms and the optimization of computational resources. SS-YOLO can also be applied to research fields, such as photosynthetic efficiency optimization and drought stress response [52], providing data-driven solutions for smart agriculture decision-making, thus promoting the development of agricultural production towards precision and intelligence.

### 4.2. Relationship Between Stomatal Parameters and Disease Resistance

For plants, stomata are not only the main channels for gas exchange but also play a crucial role in plant immune defense [45]. Pathogens often enter plant cells through stomata. Therefore, the size, density, and morphological characteristics of stomata directly affect plant disease resistance. The distribution characteristics and opening/closing ability of stomata may be important factors for plant disease defense. For example, a dense distribution of small stomata may limit pathogen invasion, whereas larger stomata may increase plant susceptibility.

This study found that there was a close relationship between stomatal parameters and disease resistance of soybean. Specifically, the DD21 consistently exhibited higher stomatal density and total area than FD9 and HX5 across all stages (healthy: 70 stomata/μm^2^, 25,764 μm^2^; asymptomatic: 59 stomata/μm^2^, 22,258 μm^2^; symptomatic: 57 stomata/μm^2^, 21,704 μm^2^), indicating a structural advantage under pathogen stress. Although stomatal density and total area decreased with disease progression, DD21 maintained higher absolute values, reflecting rapid morphological and functional adjustments that likely limit pathogen invasion while preserving physiological function.

However, the morphology and behavior of plant stomata will undergo opening and closing reactions with environmental changes. This study initially analyzed the detection of static stomata and their characteristic changes. Stomatal opening and closing are short-term responses of plants to environmental changes [53], but their morphology, distribution, and behavior will be affected by fluctuations in short-term and long-term environmental factors [34]. In future studies, we plan to extend our methodology to include time-series monitoring of stomatal aperture in living plants under biotic stress, which will complement the current structural dataset and provide a more comprehensive perspective on stomatal responses in soybean disease resistance research.

### 4.3. Limitations and Future Work

Although SS-YOLO demonstrated significant advantages in stoma detection, its practical application in real-time video monitoring in agriculture still faces multiple challenges. Firstly, in situ monitoring of living plants requires the model to maintain stability under dynamic illumination conditions and plant growth deformations [54]. However, there is a domain gap between the data trained on standard laboratory images and the complex field scenes, which affects its reliability in continuous monitoring tasks. Furthermore, SS-YOLO lacks the ability to model temporal features, such as stomatal circadian rhythms and stress responses, which limits its application value in precision agriculture decision-making.

Secondly, SS-YOLO, as a complex deep learning-based model, has high computational and memory requirements when processing large amounts of time-series image data for video monitoring in agriculture. This may become an application bottleneck on certain devices and platforms with limited computing capabilities [55]. However, with the continuous advancement of computing hardware and the optimization of deep learning techniques, SS-YOLO is expected to be widely applied in more factories and fields in the future. Broader applications can be achieved by further improving the quality of the dataset and expanding the diversity of training data. In addition, the computational demands of the model can also be effectively addressed with the design of lightweight models [56,57], further promoting the popularization and application of stoma detection technology.

## 5. Conclusions

In this study, we proposed the SS-YOLO model and quantified the changes in the stomatal characteristics of soybean leaves at different disease stages, providing a new evaluation approach for soybean disease resistance research. The results showed that there was a close relationship between stomatal parameters and soybean disease resistance, which can effectively distinguish between resistant and susceptible varieties. The main findings can be concluded as follows:

(1) SS-YOLO achieved an accuracy of 98.7% on the self-constructed soybean stoma dataset. On the public datasets, common bean, bean, and barley, SS-YOLO achieved accuracies of 98.8%, 95.1%, and 94.6%, respectively. Especially in complex backgrounds and low-contrast images, SS-YOLO significantly improved the efficiency of soybean disease resistance screening and provided strong technical support for precision breeding and agricultural production.
(2) Stoma phenotypic features, such as stoma length, width, number, and area of soybean leaves, were automatically extracted. The stomatal indices, such as stoma density, orientation, ratio of stoma area to image area, variance in area, variance in length, variance in width, uniformity, divergence, and aggregation, were calculated. These disease resistance evaluation indicators provided a new perspective for soybean disease resistance research.
(3) By exploring the relationship between stomatal characteristics and disease resistance of soybean varieties at different disease stages, the results showed that disease resistance evaluation indicators can effectively distinguish between resistant and susceptible varieties. In the self-built soybean dataset, varieties with high resistance had more stable phenotypic characteristics.

SS-YOLO exhibits strong potential for accurate stomatal detection and phenotypic parameter calculation in soybean leaves under rust infection. This enables the rapid screening of rust-resistant cultivars and supports investigations into the relationship between stomatal traits and disease resistance. In addition, by capturing stomatal changes across disease progression, the model facilitates the quantitative monitoring of infection dynamics. Beyond soybean, the approach can be extended to other crops, providing a practical tool for resistance evaluation and precision breeding.

## Figures and Tables

**Table 1:** Statistics summary of the soybean leaf stoma dataset.

Variety	Disease Stage	Number of Images	Training Set	Validation Set	Number of Stomata
DD21	healthy	138	110	28	8516
	asymptomatic	156	125	31	
	symptomatic	306	245	61	
FD9	healthy	188	150	38	7969
	asymptomatic	212	170	42	
	symptomatic	200	160	40	
HX5	healthy	232	185	47	8911
	asymptomatic	208	167	41	
	symptomatic	160	128	32	

**Table 2:** Calculation formula of stomatal parameters.

Category	Phenotypic Parameters	Symbols	Formulas	Description
basic parameters	quantity	N	N=count of bounding boxes	Total number of detected stomata
	density	D	$D=\frac{100\times N\times P^{2}}{A_{\mathrm{img}}}$	Number of stomata per unit leaf area
	area	$A_{s}$	$A_{s}=\sum_{i=0}^{n-1}\left(x_{i}y_{i+1}-x_{i+1}y_{i}\right)$	Total area of stomata
	ratio of total stoma area to image area	$Ratio_{area}$	$Ratio_{area}=\frac{A_{stomata}}{A_{image}}$	Proportion of stomata in the leaf area
	variance of stoma parameters	$Var_{A}$	$Var_{A}=\frac{1}{N}{\sum_{i=1}^{N}\left(A_{stomata}-\bar{A}\right)}^{2}$	Variability in area, width, and length
morphological parameters	length	L	L=length of major axis	Length of stoma
	width	W	W=length of minor axis	Width of stoma
	direction	θ	$\theta =\arctan\left(\frac{y_{2}-y_{1}}{x_{2}-x_{1}}\right)$	Rotation angle of the long axis of the stoma relative to the horizontal line
stomatal index	evenness	$SE_{ve}$	$SE_{ve}=\frac{\min\left(PD_{l^{\prime }}\frac{1}{N-1}\right)-\frac{1}{N-1}}{1-\frac{1}{N-1}}$	Regularity of stoma distribution
	aggregation	$SA_{gg}$	$SA_{gg}=\frac{\bar{d}}{d}$	Degree to which stoma distribution deviates from a random pattern

Note: P represents the number of pixels per 0.1 mm line. $A_{\mathrm{img}}$ represents the pixel area of the input image. $A_{stomata}$ represents the total stomatal area. $x_{i}$,$y_{i}$, represent the coordinates of stomatal vertices. $\left(x_{1},y_{1}\right)$ and $\left(x_{2},y_{2}\right)$ represent the coordinates of the two endpoints of the stomatal major axis. A minimum spanning tree was constructed to connect all stomata, minimizing the total branch length (distance between stomata). For *N* stomata, *N* − 1 branches were generated, where $PD_{l^{\prime }}$ represents the partial distance of the l-*th* branch. $\bar{d}$ represents the average of the observed nearest neighbor distances, and d represents the theoretical nearest neighbor distance. The value of ${SE}_{ve}$ ranges from 0 to 1, with values closer to 1 indicating a more uniform stomatal distribution, and values closer to 0 indicating a more clustered distribution. The $SA_{gg}$ value indicates a perfectly uniform distribution, a value equaling to 1 indicates a random distribution, and a value of less than 1 indicates clustered distribution.

**Table 3:** The ablation result of SS-YOLO over each component.

SPPF-LSKA	C2f-DLKA	Precision	Recall	mAP_50_	mAP_50:95_	FPS	Params(M)	GFLOPs
		0.925	0.850	0.936	0.464	39.42	3.01	8.12
√		0.954	0.945	0.982	0.600	36.48	4.57	8.43
	√	0.958	0.947	0.981	0.586	35.92	4.82	8.59
√	√	**0.959**	**0.950**	**0.987**	**0.854**	34.03	4.95	10.21

Note: “√” indicates that the module was used in the ablation experiment; boldface highlights the best performance.

**Table 4:** Experimental results of SOTA model comparison.

Model	Precision	Recall	mAP_50_	mAP_50:95_	FPS	Params (M)	GFLOPs
YOLOv7	0.946	0.879	0.952	0.722	37.82	3.63	8.57
YOLOv9	0.938	**0.970**	0.985	0.720	48.53	1.97	6.21
YOLOv10	0.956	0.946	0.986	0.811	44.21	2.71	7.38
ASF-YOLOv8	0.925	0.850	0.936	0.464	41.53	3.06	7.65
DynamicCov-YOLOv8	0.943	0.966	0.985	0.713	34.78	4.74	9.92
StomaYOLO	0.956	0.927	0.976	0.765	37.18	4.69	9.12
SS-YOLO	**0.959**	0.950	**0.987**	**0.854**	34.03	4.95	10.21

**Table 5:** Stoma detection results of soybean stoma on the public dataset.

Variety	Precision	Recall	mAP_50_	mAP_50:95_
Common Bean	0.949	0.977	0.988	0.573
Bean	0.905	0.909	0.951	0.610
Barley	0.902	0.898	0.946	0.751

**Table 6:** Statistics summary of soybean stomatal characteristics.

Variety	Stage	Density Stomata/μm^2^	Total Area /μm^2^	Length /μm	Width /μm	Direction /°
DD21	healthy	70	25,763.71	38.42	37.74	45.44
	asymptomatic	59	22,258.44	39.54	38.44	45.69
	symptomatic	57	21,704.21	39.31	38.88	45.24
FD9	healthy	55	17,082.91	36.03	34.45	46.18
	asymptomatic	50	15,880.96	36.51	34.67	46.41
	symptomatic	48	16,521.08	37.39	36.27	45.80
HX5	healthy	58	20,350.39	37.75	36.74	45.69
	asymptomatic	57	18,679.47	36.35	35.24	45.81
	symptomatic	54	19,484.81	38.45	37.06	46.02

## Data Availability

The data used in this study are available from the corresponding author upon reasonable request.
